# Relationship Between Magnetic Resonance T2-Mapping and Matrix Metalloproteinase 1,3 in Knee Osteoarthritis

**DOI:** 10.1007/s43465-020-00293-2

**Published:** 2020-10-29

**Authors:** Lei Shi, Kexin Wang, Jinghong Yu, Mingkai Li, Guangmei Men, Gang Ma, Xing Wang

**Affiliations:** 1 Radiology Department, The Second Affiliated Hospital of Inner Mongolia Medical University, Hohhot, 010020 Inner Mongolia China; 2 Joint Surgery, The Second Affiliated Hospital of Inner Mongolia Medical University, Hohhot, 010020 Inner Mongolia China; 3 School of Basic Medicine, Inner Mongolia Medical University, Hohhot, 010010 Inner Mongolia China

**Keywords:** T2-mapping, Articular cartilage, MMP-1,3

## Abstract

**Objective:**

To investigate the relationship between quantitative analysis of MRI (T2-mapping) and the expression of matrix metalloproteinase (MMP-1, MMP-3) in osteoarthritis of the knee joint and the role of MMP-1,3 in the pathogenesis of osteoarthritis.

**Methods:**

Thirty cases of knee osteoarthritis (KOA) patients with total knee arthroplasty (TKA) (lesion group) and 30 healthy adult volunteers (control group) were scanned with 1.5 T routine MR and T2-mapping, and their T2 values were measured and statistically analyzed. The pathological examination of the knee cartilage that was replaced during the operation and the immunohistochemical assay were used to measure the expression of MMP-1,3. The correlation between the T2 value of magnetic resonance imaging and the expression of MMP-1,3 was analyzed.

**Results:**

(1) According to the Recht grading standard for magnetic resonance, the T2 value of magnetic resonance increased significantly with the increase of cartilage degeneration. The differences in T2 values between each level and the normal group were statistically significant (*P*  < 0.05). (2) The T2 value of magnetic resonance imaging increased with the severity of the cartilage degeneration pathological Mankin grading, and the difference was statistically significant (*P*  <  0.05). (3) The expression of MMP-1,3 increased with cartilage degeneration. (4) The T2 value and the expression of MMP-1 in cartilage showed a linear trend. The result of Spearman correlation analysis showed that the expression of MMP-1,3 increased as the cartilage T2 value increased. There was a positive linear correlation between the two.

**Conclusion:**

The T2 value of magnetic resonance increased with the degeneration of KOA cartilage. The expression of MMP-1,3 increased with the severity of articular cartilage destruction. The T2 value of KOA magnetic resonance was positively correlated with the expression of MMP-1,3.

## Introduction

KOA is a common chronic progressive arthropathy in the clinic. The etiology of KOA was complicated and the pathogenesis is unknown. The disability rate of knee osteoarthritis in the late stage is high. The mainstream treatment of knee osteoarthritis in the late-stage is Arthroplasty of the whole knee. The disease brings a serious economic burden to the family and society [1]. Therefore, early diagnosis and effective treatment on the specific proteins have always been the hot spot of clinical research.

The major pathological change of OA is the degeneration of articular cartilage. Imbalance between the production and degradation of the extracellular matrix of articular cartilage is the basic step that causes degeneration of articular cartilage [2, 3]. Matrix metalloproteinases (MMPs) plays an important role in the degeneration and process of OA cartilage, considered to be the key enzyme for the degradation of OA cartilage matrix, high expression in the cartilage of patients with OA. It is believed the MMPS expression indicates the severity of OA [4, 5]. Meanwhile, the physiological MRI of cartilage, which is emerging in recent years, can not only clearly display the morphological changes of cartilage, but also detect the content and structural changes of cartilage biochemical ingredients sensitively, which indicates the severity of OA and provide a new approach for the early non-invasive quantitative diagnosis of OA. T2-mapping is currently considered to be the most effective non-invasive technique for the evaluation of articular cartilage. T2 value of cartilage in the degeneration status is higher than that of normal cartilage, and T2 value of magnetic resonance imaging increased with the degree of degeneration of knee articular cartilage [6–9], which is also a focused research. As far as we know, the correlation between MRI results of osteoarthritis and the expression of cartilage MMPS has not been reported.

The purpose of this study is to assess the degree of articular cartilage degeneration in KOA patients by the non-invasive sensitive physiological MRI-T2-mapping, detecting the expression of MMP-1,3 in cartilage, to study the correlation between T2-mapping and MMP-1,3 expression. Further, the MRI results were used to evaluate the pathophysiological changes of OA, contributed to the diagnosis OA in the early stage, treated the patients of OA in different pathophysiological stages specifically and were used for the evaluation of the curative effect in the future. The non-invasive sensitive physiological MRI-T2-mapping is of great significance in delaying the progression of the disease, preventing disability and improving the therapeutic effect of OA.

## Materials and Methods

### General Information

A total of 30 patients with TKA in Second Affiliated Hospital of Inner Mongolia Medical University from October 2016 to October 2017 were selected, including 4 males and 26 females. There were 13 cases of injury on the left knee and 17 cases of injury on the right knee. The average age was 63.45. A total of 30 healthy adult volunteers (15 cases of the left knee, 15 cases of the right knee) were selected as the control group (BMI 18.5–23.9), including 11 males and 19 females. The average age was 32.60 years. Exclusion criteria: knee pain, trauma, history of surgery, and history of joint disease.

#### Exclusion Criteria

(1) Patients with serious primary diseases, such as cardiovascular and cerebrovascular diseases, high blood pressure or complications arising from the digestive system, hemopoietic system, liver and kidney. (2) Psychotic patients, individuals with the allergic constitution, or susceptible to co-infection and bleeding; Women who are pregnant, breastfeeding or in their menstrual period. (3) Secondary arthritis, such as rheumatoid arthritis, rheumatoid arthritis, ankylosing spondylitis, gouty arthritis, idiopathic arthritis, post-infection arthritis, metabolic bone disease, post-traumatic arthritis. (4) Pain caused by non-osteoarthritis, such as fibromyalgia or other joint diseases. (5) Individuals who cannot cooperate with the examination or other unsuitable cases for MRI scan. For example, patients with claustrophobia or metal implants in their body. (6) Patients who have had treatment interventions such as joint cavity injections. (7) BMI is outside the range of 18.5–23.9 (excluding the influence of weight).

### MRI Examination

GE 1.5T MR-360 Magnetic resonance imaging system (MRI) instrument was used in an 8-channel special coil for the knee joint. All the patients signed informed consent before the examination. All patients were instructed to minimize knee movement and sit still before MRI scanning. Patient was laid supine with the long axis of the knee joint parallel to the long axis of the scanning bed to minimize the effect of the magic angle effect.

Conventional sagittal FSE T1WI scan parameters were preset as: TR = 226 ms, TE = 19.4 ms, slice thickness: 3 mm, FOV: 16 mm × 16 mm, matrix: 256 × 192, NEX = 2; sagittal FSE-fs-T2WI scan parameters are: TR = 2477 ms, TE = 109.8 ms, layer thickness 3 mm, FOV: 16 mm × 16 mm, matrix: 192 × 192, NEX = 2.

T2-mapping imaging was a multi-echo sequence FSE sagittal scan. Parameters as follows: the TR 1534 ms, TE 12.1 ms/24.3 ms/36.4 ms/45.5 ms/60.7 ms/72.8 ms/85 ms/97.1 ms, slice thickness was 4.0 mm, slice gap was 0.2 mm, FOV 16 mm × 16 mm, matrix 256 × 224, NEX = 1, the scanning time was 5.5 min.

### Image Post-Processing and Image Analysis

When the scan was completed, all the original image data was transferred to workstation ADW4.6 for post-processing. The Functool 9.4.05 software was used to obtain the T2-mapping pseudo color image, and a fusion T2 map was obtained in combine with the sagittal FSE T1WI scan. The maximum weight-bearing areas of the medial femoral condyle, lateral condyle, medial tibial platform, and lateral platform were selected as the region of interests (ROI), and the ROI were manually selected on the T2 map. The T2 value was measured form the selected ROI. The T2 value of each cartilage ROI was measured, and each ROI area was measured 3 times, and the average value was obtained for further analysis.

The image was analyzed by two experienced radiologists with more than 20 years of experience in the diagnosis of musculoskeletal imaging. Recht grading criteria were used for the MRI diagnostic grading of cartilage [10]: Class I: articular cartilage is structurally complete, but the thickness might reduce, and the surface is smooth; Class II: cartilage layering disappears, locally low signal appears, the cartilage surface is still smooth; Class III: cartilage surface is mild or moderately irregular, and the articular cartilage is defective. However, the thickness is less than half of normal values; Grade IV: severe defects on cartilage surface, articular cartilage defects exceed half of the normal thickness, but not completely exfoliated; Grade V: complete cartilage exfoliation, severe defect, exposed subchondral bone, may have signal changes at the subchondral bone.

### Pathological and Imunohistochemistry Assays

The medial and lateral condyle of the femur and the medial and lateral of the tibial plateau from the patients with KOA were used for sampling, decalcification, embedding and section. All specimens were pathological HE stained to evaluate the histological changes of cartilage and the expression of MMP-1,3 in articular cartilage was detected by immunohistochemistry. HE stained slides were reviewed by two senior pathologists and graded according to Mankin grading [11]. Immunohistochemical results were evaluated by Pelletier et al. [12] method. Image-proplus6.0 was used for semi-quantitative analysis of positive cells. The percentage of stained versus total cells was used to evaluate the expression of MMP-1,3 in cartilage tissue.

### Statistical Methods

SPSS 17.0 statistical software was used to analyze data, one-way ANOVA was used when the data met normality and the homogeneity of variance. LSD test was used for pairwise comparison between groups, otherwise, a non-parametric Kruskal–Wallis test and Mann–Whitney test were adopted, Spearman correlation analysis was used to analyze the correlation between the expression of cartilage MMP-1,3 and the T2 value of MRI in cartilage. *P* < 0.05 was considered statistically significant.

## Results

### T2-Mapping Imaging of Knee Cartilage and the MRI Grading of T2 Value

**Fig. 1:**
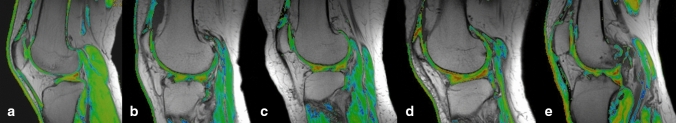
T2 mapping MRI grading pseudocolor of OA articular cartilage. **a** Shows that the cartilage of the knee joint is smooth and continuous, and the articular cartilage has a uniform yellow-green color, mainly green. **b** Shows OA articular cartilage grade I. The articular cartilage surface is smooth, the cartilage thickness is uneven, the yellow-green color scale is uneven, and the spot-like red color scale is visible. **c** Shows grade II: frizziness on the surface of cartilage, slightly narrowing of joint space, and irregularly shaped red gradations. **d** Shows grade III: severe irregular surface contours of articular cartilage, cartilage defects, and mixed color unevenness in cartilage. **e** Shows grade IV: full-thickness cartilage defect, exfoliation, narrowing of the joint space, formation of a large number of osteophytes around the bone, and exposure of subchondral bone with changes in bone signal

The results of MRI T2-mapping imaging of normal control group and patients with KOA (Fig. 1) and Recht grading (Table 1) between normal adults and 30 patients with KOA showed that the T2 values of articular cartilage in the medial and lateral condyle of the femur and the medial and lateral of the tibial plateau in patients were higher than those in the normal group. The T2 values increased with the rising of the MRI grade and the difference was dramatically between the normal group and the patient group in the high Recht grading level (Table 2).

**Table 1:** MRI classification of cartilage in various areas of the knee joint with OA (unit: a piece)

Site	Grade I	Grade II	Grade III	Grade IV
Medial femur	2	4	9	16
External femur	6	5	12	7
Medial tibial plateau	3	5	11	14
Lateral tibial plateau	4	4	10	8

**Table 2:** T2 values of cartilage in different parts of knee joint ($\overline{X}$  ± * S*)

Group	Medial femur	External femur	Medial tibial plateau	Lateral tibial plateau
Normal	37.66 ± 2.14	36.66 ± 1.74	36.72 ± 2.43	37.09 ± 1.82
Grade I	40.61 ± 1.57	38.71 ± 1.79	39.72 ± 1.88	39.49 ± 1.28
Grade II	38.86 ± 1.49	40.59 ± 2.92	43.46 ± 1.03a	41.69 ± 1.68a
Grade III	50.96 ± 5.10abc	46.58 ± 4.19abc	49.07 ± 4.78abc	46.43 ± 2.94abc
Grade IV	53.11 ± 3.64abcd	49.53 ± 7.06abc	55.37 ± 4.65abcd	52.10 ± 4.71abcd

The comparison between a and normal, *P* < 0.05, The comparison between b and grade I, *P* < 0.05, The comparison between c and grade II, *P* < 0.05, The comparison between d and grade III, *P* < 0.05

### The Relationship Between T2-Mapping of OA Articular Cartilage and the Mankin Grading of Cartilage Histopathology

**Fig. 2:**
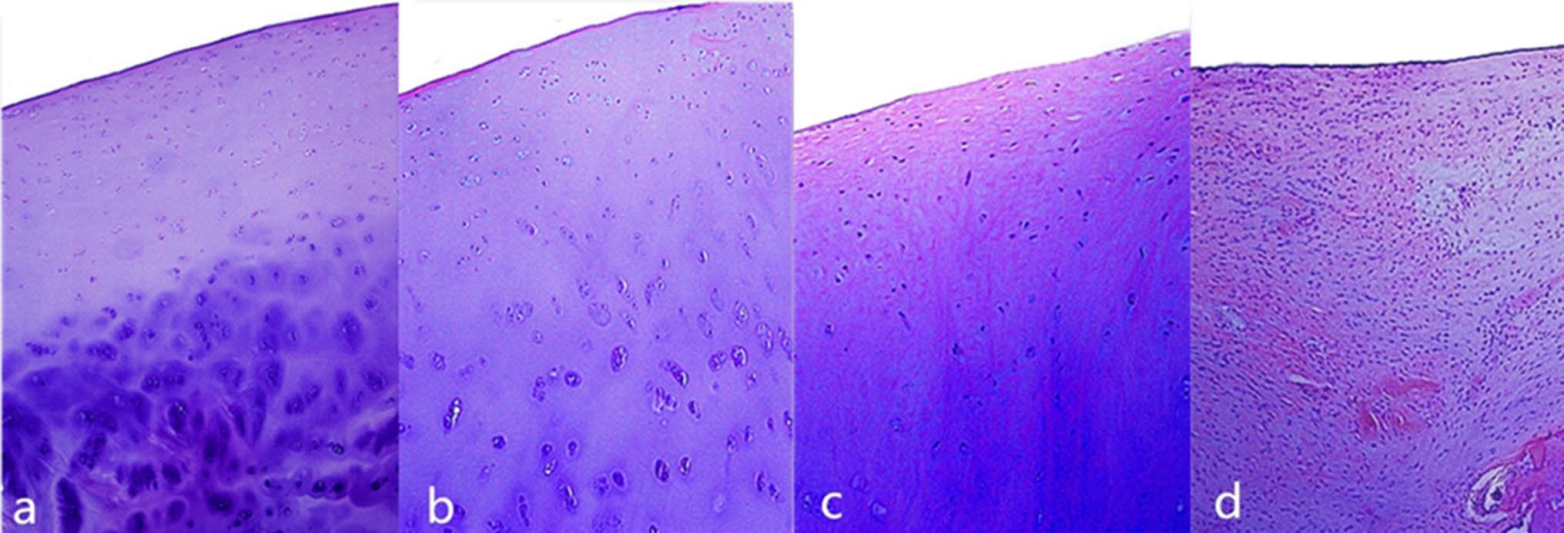
Pathological grade of knee joint cartilage he (× 100). Grade I–IV degeneration of the cartilage of the knee joint: the chondrocyte matrix is unevenly stained and the chondrocytes are disordered; the cartilage surface is rough, the normal structure of the cartilage surface is destroyed, and the matrix is unevenly stained; the chondrocytes are suddenly reduced and the structure is disordered; obviously the cartilage structure was damaged and the chondrocytes were necrotic

The comparison table between the T2 value of KOA cartilage and Mankin pathological classification control (Fig. 2) in the 30 patients was shown in Table 3. The T2 value increased with the rising of pathological grading degree in cartilage degeneration. There were statistically significant differences in T2 values of I, II, III, IV level and normal group (*P* < 0.05). The difference between high grade and the low grade was statistically significant (*P* < 0.05).

**Table 3:** Degenerative cartilage T2 values with pathological grade control

Grading	The number of lesions subregio (a piece)	T2 values (ms)	Standard
Grade I	15	39.37	1.62
Grade II	18	41.24	2.47
Grade III	42	48.13	4.55
Grade IV	45	53.08	5.01

### Expression of MMP-1,3 in OA Articular Cartilage and Correlation Analysis Between the Expression and T2 Value of MRI

**Fig. 3:**
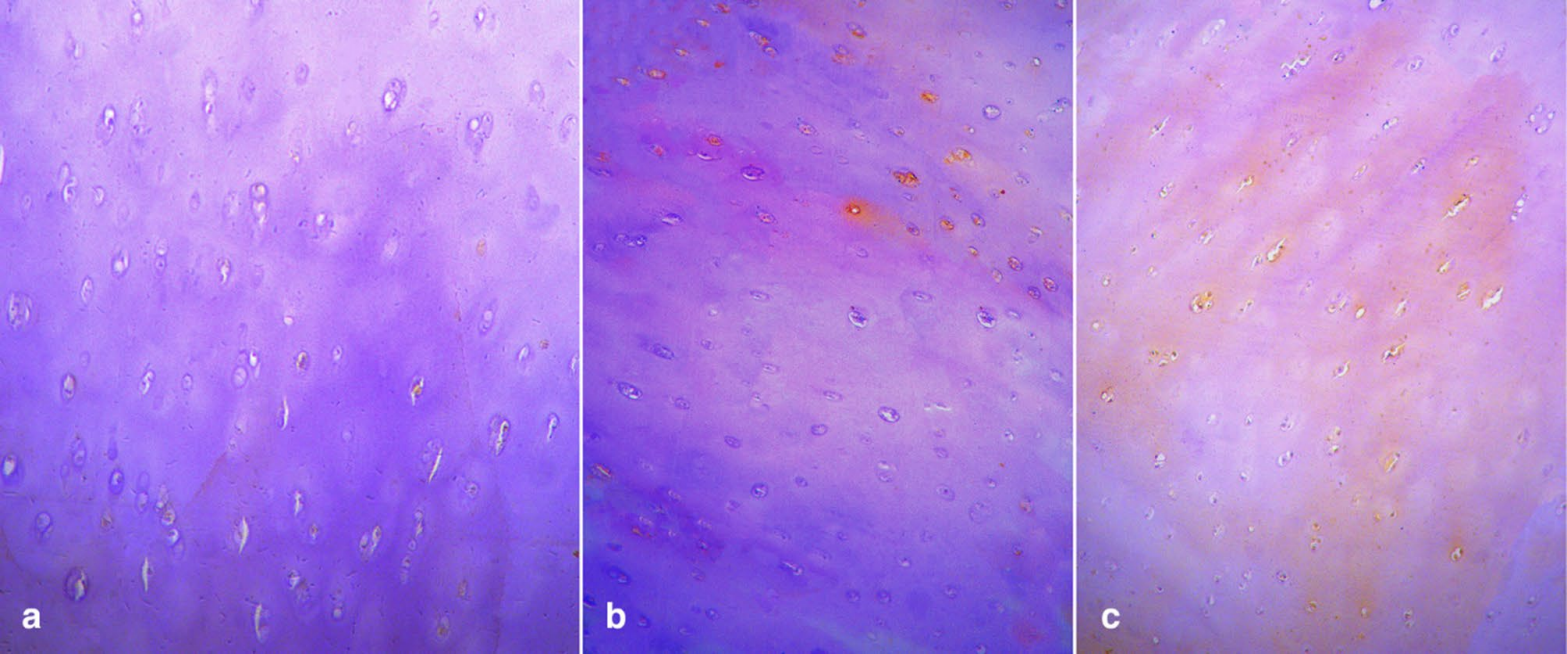
MMP-1 immunohistochemical staining of knee cartilage (× 100).With the increase of knee cartilage degeneration, brown cells increased and the expression of MMP-1 increased significantly

The results of immunohistochemical assay for MMP-1 (Fig. 3) showed that the positive rate of MMP1 in different parts of knee articular cartilage at different levels was different. The expression increased with the rising of pathological grading degree in cartilage degeneration. The difference between high and low grades was dramatical with pathological grading (Table 4).

**Table 4:** The expression of MMP1 in different MR grades of knee OA articular cartilage (%) ($\overline{{\text{X}}}$ ± *S*)

Group	Medial femur	External femur	Medial tibial plateau	Lateral tibial plateau
Grade I	4.23 ± 0.93	3.48 ± 0.81	3.15 ± 0.69	2.63 ± 0.78
Grade II	7.53 ± 1.22	6.34 ± 0.82b	8.07 ± 1.67b	6.01 ± 0.58
Grade III	15.62 ± 2.88bc	13.49 ± 3.37bc	13.49 ± 3.19bc	13.19 ± 2.41bc
Grade IV	26.12 ± 4.97bcd	26.26 ± 5.99bcd	25.95 ± 5.16bcd	26.77 ± 5.13bcd

The comparison between b and grade I, *P* < 0.05, The comparison between c and grade II, *P* < 0.05. The comparison between d and grade III, *P* < 0.05

**Fig. 4:**
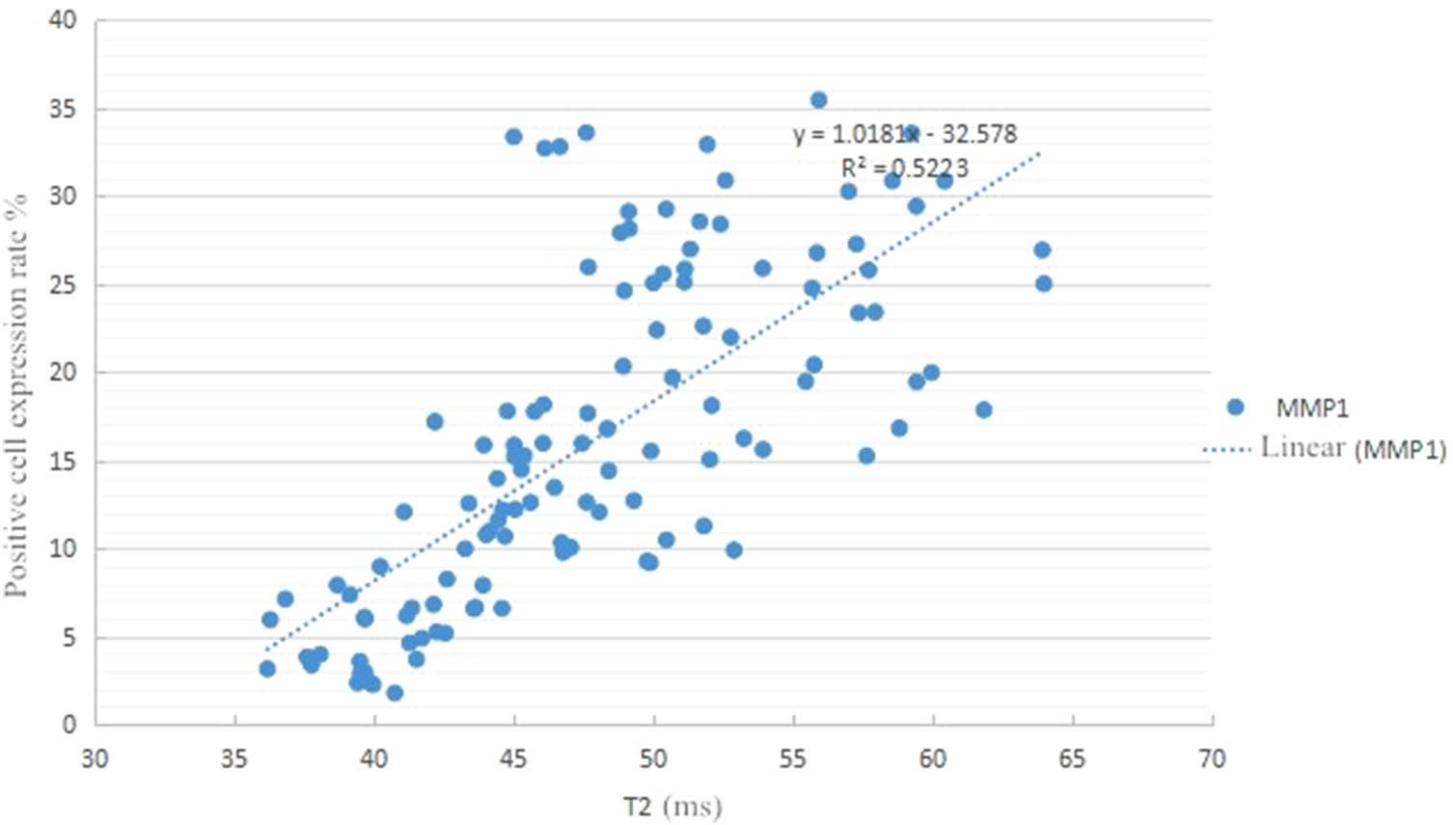
Scatter plot of T2 value and MMP1 expression in OA knee articular cartilage

A scatter diagram of the T2 value for OA cartilage and the positive rate of MMP-1 (Fig. 4) showed that the T2 value and the expression of MMP-1 of cartilage presented a linear tendency. Spearman correlation analysis indicated that MMP-1 expression increased with the rising of T2 value. The expression of MMP-1 was positively linearly correlated with the T2 value.

**Fig. 5:**
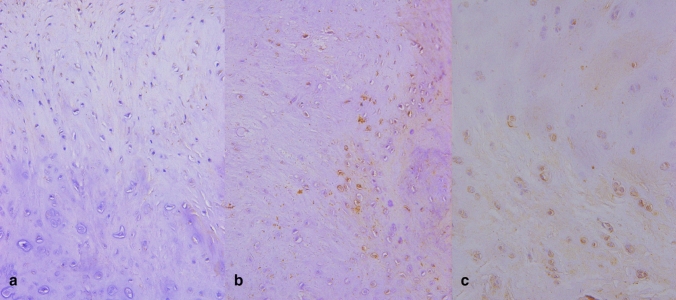
MMP-3 immunohistochemical staining of knee cartilage (× 100).The expression of MMP-3 increased with the degree of degeneration of cartilage in the knee joint, and it was found that the expression of MMP-3 was higher in the lower and deep layers of cartilage

**Fig. 6:**
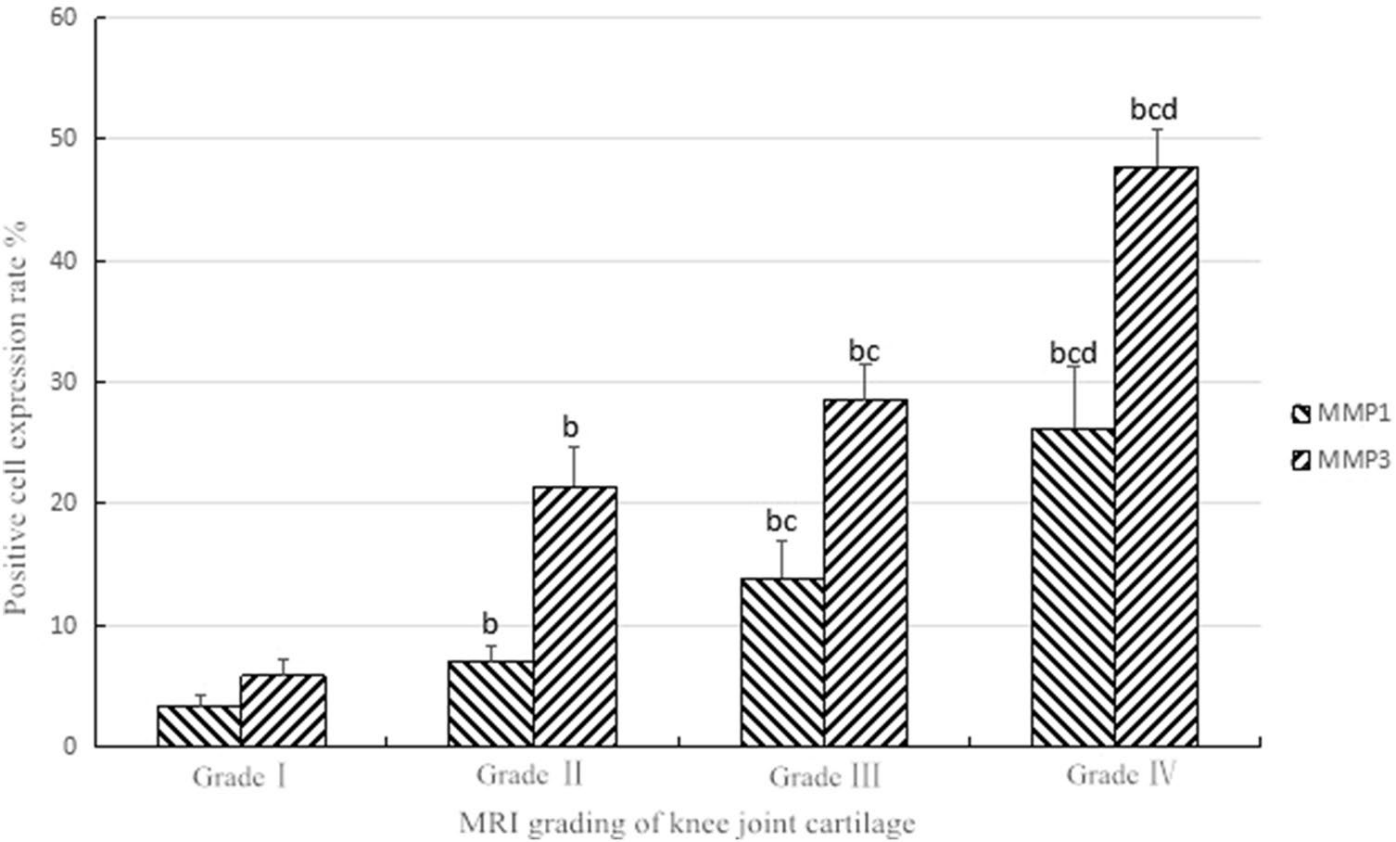
The expression of MMP1, MMP3 in various grades.The comparison between b and grade I, *P* < 0.05, The comparison between c and grade II, P < 0.05, The comparison between d and grade III, *P* < 0.05

The results of immunohistochemical staining of MMP-3 (Fig. 5) showed that the expression rates of MMP-3 positive cells the medial and lateral condyle of the femur and the medial and lateral of the tibial plateau were various in different MRI grades of OA. The expression of MMP-3 was increased with the rising of MRI grades. The difference between high and low grades was dramatical. There were statistically significant differences between III, IV levels and I, II levels in the medial and lateral condyle of the femur (*P* < 0.05). The comparison between III, IV levels and I, II levels in the medial and lateral of the tibial plateau was statistically significant (*P* < 0.05), as shown in Table 5 (Fig. 6).

**Table 5:** The expression of MMP-3 in different MR grades of knee OA articular cartilage (%) ($\overline{{\text{X}}}$ ± *S*)

Group	Medial femur	External femur	Tibial medial plateau	Lateral tibial plateau
Grade I	8.14 ± 0.84	6.33 ± 0.62	5.15 ± 0.68	4.46 ± 0.96
Grade II	nn19.35 ± 1.70b	19.94 ± 2.95b	24.67 ± 2.98b	21.44 ± 1.84b
Grade III	27.91 ± 3.16b	26.09 ± 2.28bc	30.19 ± 2.13b	30.22 ± 1.59b
Grade IV	46.66 ± 2.46bcd	43.56 ± 2.13bcd	49.77 ± 1.59bcd	50.16 ± 0.78bcd

The comparison between b and grade I, *P* < 0.05. The comparison between c and grade II, *P* < 0.05. The comparison between d and grade III, *P* < 0.05

The positive rates of MMP-1 and MMP-3 showed an upward trend with the increase of the pathological grading, and the difference between the different grades by Kruskal–Wallis test was statistically significant (*P* < 0.05). Pairwise comparison of Mann–Whitney test between different grades showed statistical significance (*P* < 0.05). As shown in Fig 6.

## Discussion

As the clinical course of OA is complicated and clinical samples of OA are difficult to obtain, early detection, diagnosis and intervention in different pathological stages have always been the focus of OA clinical research. There are many studies on imaging and pathophysiology of OA, however, most of the studies are reports on single technology [13]. Compared with previous studies, our study has contributed to different aspects of the theme outlined: (1) We not only reported the T2-mapping images of OA patients but also investigated the pathologic results of the knee cartilage that was replaced during the operation and measured the expression of MMP-1,3 of the cartilage by the immunohistochemistry. (2) A controlled study was conducted on T2 value of knee cartilage and histopathology of corresponding cartilage. (3) The expression of MMP-1,3 in the cartilage at different MRI levels in the OA group was analyzed. (4) Correlation analysis was performed between the T2 value of KOA cartilage and the expression of MMP-1,3.

Nowadays, lots of studies confirm that T2-mapping is an effective approach for noninvasive evaluation of cartilage, which presents the T2 value of cartilage and indicates the variation of collagen content, the alignment direction of collagen and the variation of water content in articular cartilage. Some studies [14] indicate that the result of T2-mapping was correlated with MRI grading of the articular cartilage injury. T2-mapping sequence presents early damage of knee cartilage without obvious morphological changes, and T2-mapping could be used to diagnose the damage of knee articular cartilage in an early stage. The results of our study show the abnormal signals, the thinning thickness of cartilage, loss of cartilage and other abnormal changes are gradually intensified with the increase of MRI grade in OA group by T2-mapping. The T2 value of every ROI in OA group is higher than that in the control group, and T2 value is significantly increased with the rising of MRI grade in OA articular cartilage degeneration. The T2 values increased significantly with the rising degree of histologically cartilage degeneration (Mankin classification). The results are consistent with other reports, suggesting T2-mapping is available for presenting the severity of OA cartilage degeneration and the early change of OA noninvasively.

On the other hand, some studies indicate matrix metalloproteinases (MMPs) play an important role in the degradation of cartilage matrix in the occurrence and development of OA [15]. MMPs are the generic terms of a group of endopeptidases that are structurally highly homologous and contain Zn^2+^. They degrade most of the extracellular matrix proteins and are considered to be the key enzymes for the degradation of OA cartilage matrix [16]. The expression of MMPs in synovial and cartilage tissues of normal people is low, but it is highly expressed in those of OA patients [17]. MMP-1, MMP-3 and MMP-13 have been the main topic of research [18]. It is reported that MMP-1 is crucial for the specific degradation of articular cartilage matrix collagen type II, the degradation effect of MMPs on type II collagen is relying on MMP-1 [19]. Wu et al. [20] reported the expression of MMP-1 was significantly increased in OA articular cartilage. The degree of cartilage degeneration was positively correlated with the increased expression of MMP-1. MMP-3 degrades a variety of matrix collagen directly and plays an important role in the degradation of proteoglycan, which maintains a stable efficiency in the development of OA [21]. MMP-3 activates MMP-1 and promotes the damage of cartilage by a synergistic with other members of the MMPs family [22]. Lv et al. [23] reported the level of MMP-3 expression in both the synovial membrane and chondrocytes of the OA rabbit models was high, and the expression of MMP-3 increased in a time-dependent manner. The results of immunohistochemistry suggest that the expression of MMP-1,3 in OA cartilage increased with the severity of cartilage degeneration and with the rising of MRI classification. The expression of MMP-1,3 indicates the level of OA cartilage degeneration degradation. The MMP-1 is expressed mainly on the superficial layers and middle-upper layers of cartilage, however, MMP-3 is expressed in the deep layers of cartilage. Different expressions of MMP-1 and MMP-3 in various cartilages indicate different parts of the degenerated cartilage.

Detecting the expression of MMPs in vivo is difficult, as sampling OA cartilage in the clinic is not easy and not popular. A non-invasive detecting alternative to the detection of MMPs in cartilage is necessary. OA is mainly caused by the degeneration of articular cartilage pathologically. The basic step of the degeneration of articular cartilage is the degradation of extracchondral matrix (containing water, proteoglycan and collagen). MMPs play a major role in the degradation of the cartilage matrix as the expression is increased in OA patients. MRI T2-mapping is sensitive to the changes of water and collagen in the cartilage matrix. T2 value is also increased in OA patients. As there may be a relationship between the T2 value and the expression of MMPs in cartilage, the correlation analysis between the T2 value of cartilage and the expression of MMP-1,3 in OA group was conducted. The results showed there was a positive linear correlation between the T2 value and the expression of MMP-1,3 of OA cartilage. The expression of MMP-3 increased more than that of MMP-1, which suggests T2 value could not only indicate the severity of OA cartilage degeneration but also suggest the level of MMP-1,3 in the degradation of cartilage. The correlation between the T2 value and the expression of MMP-1,3 could be used for selecting specific MMPs antagonistic drugs and treatment regimens based on the expression levels of MMPs and monitoring the effect of treatment. MMP-1,3 are expected to be early monitoring indicators for OA, help an accurate diagnosis of OA, reflect the of progression OA and treat early OA. This will provide benefits for future diagnosis, clinical monitoring and follow-up of clinical OA.

At present, there are reported clinical cases of applying MMPS inhibitors in treating OA [24, 25], the underlying mechanism of which is to inhibit the inflammation of articular cartilage and the degradation of cartilage by reducing the production of MMPS, thereby inhibiting inflammation and degradation of articular cartilage. Hence, achieving better protection of chondrocytes, improved outcome of clinical symptoms; helping with the ease and repair of inflammation. However, it is worth to note that the stated curative effect still needs the validation from large-scale images or experimental data. The preliminary results of this study indicate that the T2 value can be used as an indicator to monitor the content of MMPs and the degree of articular cartilage damage, and also has been demonstrated for its good sensitivity and specificity. Direct measurement of MMPs with living cartilage is the difficulty; however, the prospect of utilizing T2 value as an objective and quantitative imaging reference for evaluating and monitoring the efficacy of MMPS inhibitors is acknowledged in our study. Promisingly, T2-mapping technology could be applied as an imaging method for early diagnosis, quantitative evaluation, and real-time monitoring of OA, and provide evidence-based support for early clinical intervention.

Deficiencies of this study: (1) The overall sample size is small; (2) Most patients in the OA group are a late stage of OA sufferers with arthroplasty of the whole knee, the samples with early stage of OA is few. (3) T2-mapping was conducted for a long time by 1.5T MRI scan;(4) There is no precise correspondence between the deep/superficial layer of cartilage and the T2 value of cartilage as well as the immunohistochemical results of MMPS-1,3. It is necessary to enlarge the sample size or obtain more experimental data by animal experiments.

The T2 value obtained by MRI T2-mapping technology is positively correlated with the expression of MMP-1,3, a key enzyme in cartilage matrix degradation, and the T2 value increases with the severity of KOA articular cartilage disease. It can reflect the expression level of MMPS in articular cartilage and can provide objective imaging evidence for the efficacy of MMPs antagonists. T2-mapping technology has shown its prospects of clinical application in the entire pathophysiology of OA patients, and is expected to become a non-invasive objective imaging index for early detection, accurate diagnosis, or be used as a reference for monitoring and follow-up studies post MMPs antagonists treatment.
