# Correction to: Relationship Between Magnetic Resonance T2-Mapping and Matrix Metalloproteinase 1,3 in Knee Osteoarthritis

**DOI:** 10.1007/s43465-020-00310-4

**Published:** 2021-04-19

**Authors:** Lei Shi, Kexin Wang, Jinghong Yu, Mingkai Li, Guangmei Men, Gang Ma, Xing Wang

**Affiliations:** 1 Radiology Department, The Second Affiliated Hospital of Inner Mongolia Medical University, Hohhot, 010020 Inner Mongolia China; 2 Joint Surgery, The Second Affiliated Hospital of Inner Mongolia Medical University, Hohhot, 010020 Inner Mongolia China; 3 School of Basic Medicine, Inner Mongolia Medical University, Hohhot, 010010 Inner Mongolia China

## Correction to: Indian Journal of Orthopaedics 10.1007/s43465-020-00293-2

The original version of this article unfortunately contained some mistakes. Some figures were incorrect and interchanged respectively. The correct assignment of figures is as follows: Figs. 2, 4 and 6 were replaced by new figures. The previous Fig. 2 in the article is actually Fig. 3, the previous Fig. 3 in the article is actually Fig. 5, and the previous Fig. 5 was deleted. In addition, reference [3] was presented incorrectly. The correct figures and reference are given below.

**Fig. 2:**
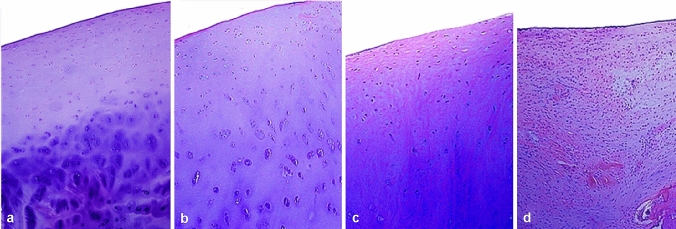
Pathological grade of knee joint cartilage he (× 100). Grade I–IV degeneration of the cartilage of the knee joint: the chondrocyte matrix is unevenly stained and the chondrocytes are disordered; the cartilage surface is rough, the normal structure of the cartilage surface is destroyed, and the matrix is unevenly stained; the chondrocytes are suddenly reduced and the structure is disordered; obviously the cartilage structure was damaged and the chondrocytes were necrotic

**Fig. 3:**
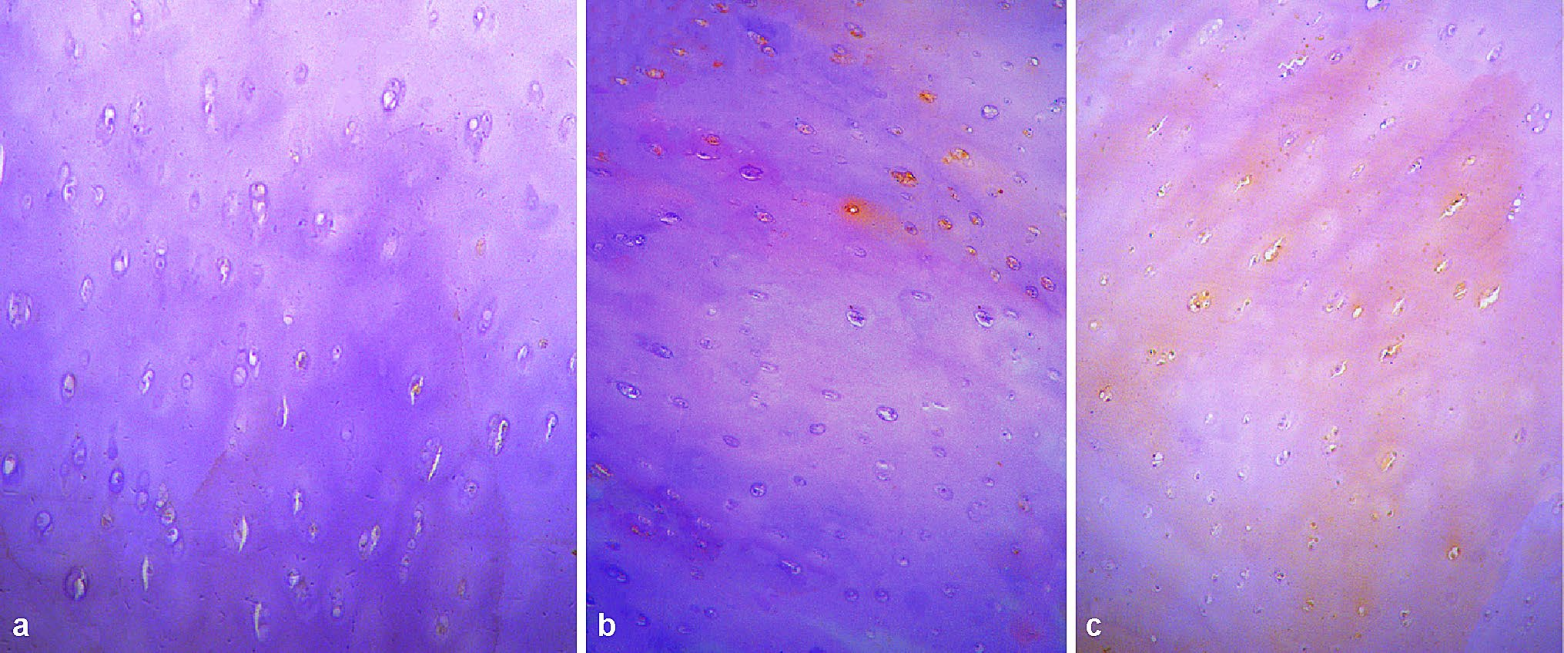
MMP-1 immunohistochemical staining of knee cartilage (× 100). With the increase of knee cartilage degeneration, brown cells increased and the expression of MMP-1 increased significantly

**Fig. 4:**
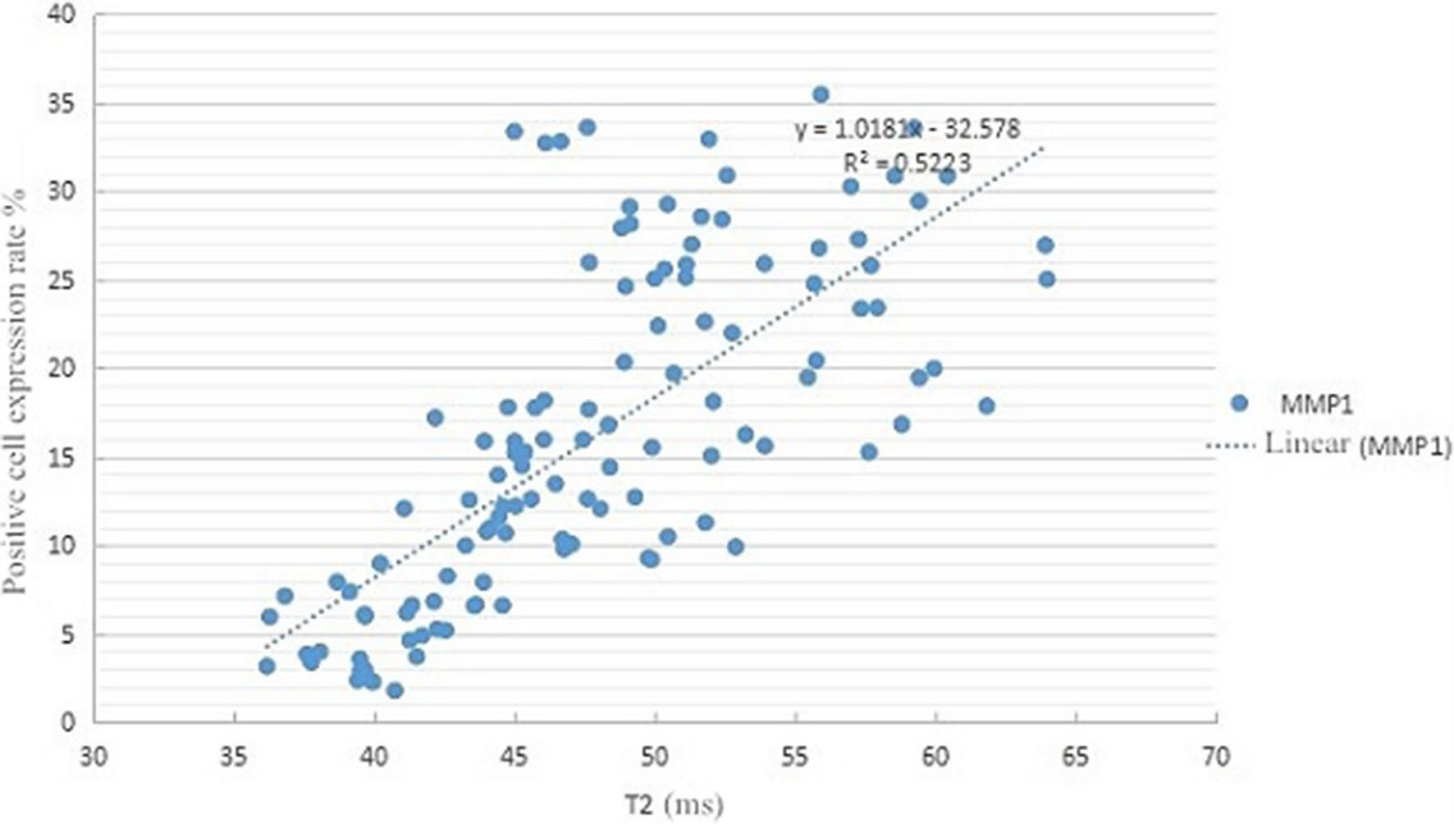
Scatter plot of T2 value and MMP1 expression in OA knee articular cartilage

**Fig. 5:**
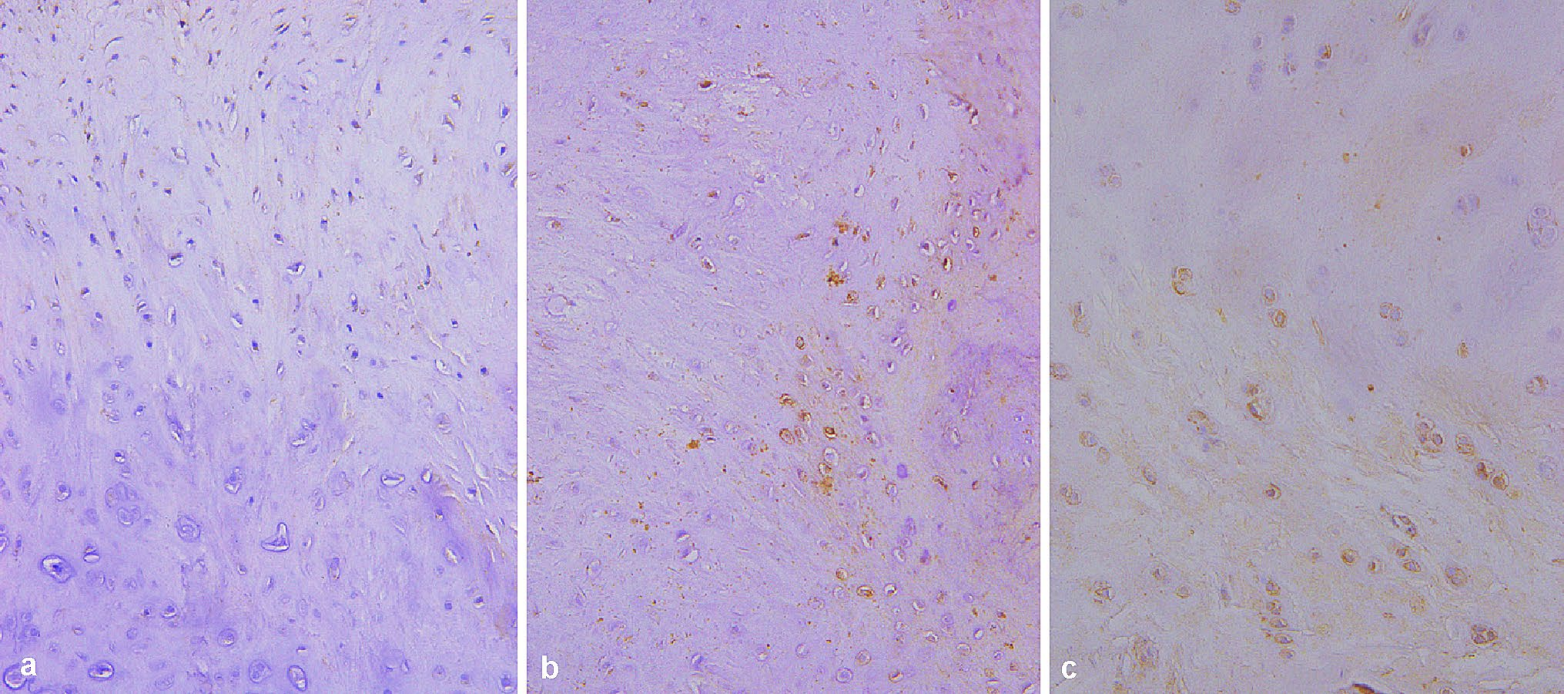
MMP-3 immunohistochemical staining of knee cartilage (× 100). The expression of MMP-3 increased with the degree of degeneration of cartilage in the knee joint, and it was found that the expression of MMP-3 was higher in the lower and deep layers of cartilage

**Fig. 6:**
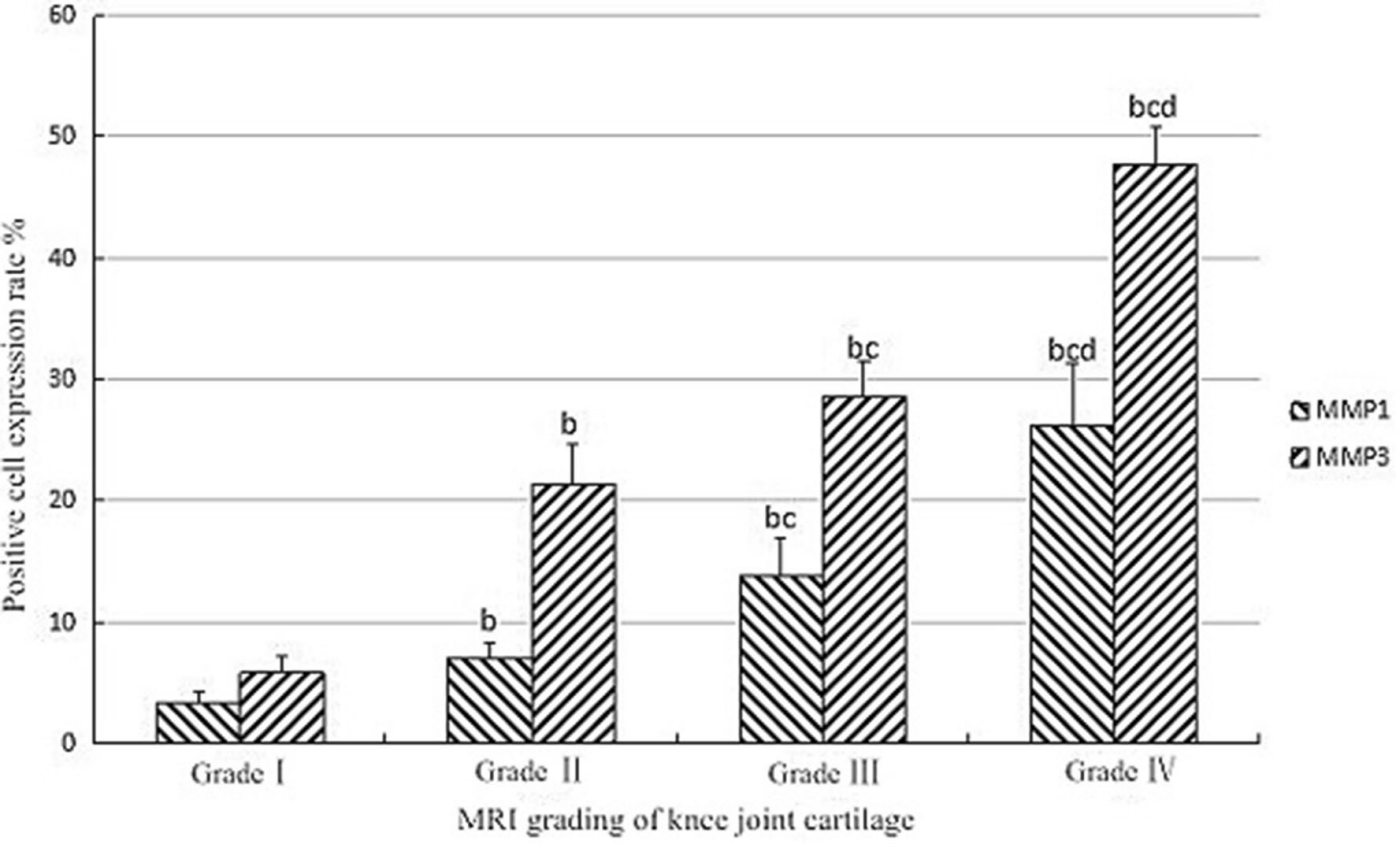
The expression of MMP1, MMP3 in various grades. The comparison between b and grade I, *P* < 0.05, The comparison between c and grade II, *P* < 0.05, The com-parison between d and grade III, *P* < 0.05

The original article has been corrected.

## References

[3] Chen D, Shen J, Zhao W (2017). Osteoarthritis: toward a comprehensive understanding of pathological mechanism. Bone Res.

